# FAIRification of health-related data using semantic web technologies in the Swiss Personalized Health Network

**DOI:** 10.1038/s41597-023-02028-y

**Published:** 2023-03-10

**Authors:** Vasundra Touré, Philip Krauss, Kristin Gnodtke, Jascha Buchhorn, Deepak Unni, Petar Horki, Jean Louis Raisaro, Katie Kalt, Daniel Teixeira, Katrin Crameri, Sabine Österle

**Affiliations:** 1 Personalized Health Informatics Group, SIB Swiss Institute of Bioinformatics, 4051 Basel, Switzerland; 2 Trivadis — Part of Accenture, 4051 Basel, Switzerland; 3 Health Informatics and Data Privacy Group, Biomedical Data Science Center, 1010 Lausanne University Hospital, Lausanne, Switzerland; 4 Clinical Data Platform Research, Directorate of Research and Education, Zurich University Hospital, 8091 Zurich, Switzerland; 5 DSI - Data Group, Geneva University Hospital, 1205 Geneva, Switzerland

**Keywords:** Health care, Medical research

## Abstract

The Swiss Personalized Health Network (SPHN) is a government-funded initiative developing federated infrastructures for a responsible and efficient secondary use of health data for research purposes in compliance with the FAIR principles (Findable, Accessible, Interoperable and Reusable). We built a common standard infrastructure with a fit-for-purpose strategy to bring together health-related data and ease the work of both data providers to supply data in a standard manner and researchers by enhancing the quality of the collected data. As a result, the SPHN Resource Description Framework (RDF) schema was implemented together with a data ecosystem that encompasses data integration, validation tools, analysis helpers, training and documentation for representing health metadata and data in a consistent manner and reaching nationwide data interoperability goals. Data providers can now efficiently deliver several types of health data in a standardised and interoperable way while a high degree of flexibility is granted for the various demands of individual research projects. Researchers in Switzerland have access to FAIR health data for further use in RDF triplestores.

## Introduction

A large amount of data is generated daily by a variety of health care providers and individuals, representing potentially valuable information for personalised health research, public health, improvement and monitoring of health care quality. Health data, from routine clinical data and imaging data to sensor and multiomics, are often highly heterogeneous and stored in different databases (or silos). The meaning of the data is often hidden behind local formats or proprietary standards from electronic health record (EHR) vendors^1,2^. This fragmentation and diversity make it time-consuming to access, combine and use data from various hospitals, laboratories, or other data providers. Often, the intended meaning of a data resource is difficult to understand, and merging data from different sources is extremely challenging due to the lack of common data dictionaries and the variety of ways in which metadata are represented. Several “common” data models for clinical data representation have been developed^3–5^ to standardise recorded information, but there are still problems with sharing and reusing clinical resources^6^. These data models are usually developed to serve a specific purpose or process and can be difficult to extend beyond their original scope. For specific research projects, especially in the area of personalised health but also for other use cases, such as quality of care, regulatory and clinical trials, public health, and patient safety, which require specific contextual information, these models are insufficient and offer too little flexibility.

The Swiss Personalized Health Network (SPHN, https://sphn.ch/) initiative aims to build a sustainable and federated infrastructure for the use and exchange of health-related data for research purposes in a FAIR manner^7^. The FAIR principles, introduced in 2016 by Wilkinson *et al*.^8^, constitute practical guidelines for better representing digital information to make it findable, accessible, interoperable and reusable. The goal of the FAIR principles is to agree on and anchor common practices among data providers so that users can more easily reuse the data to their full potential by end users. Although the implementation of these principles can be a complex task^9^, many scientific projects have already taken the challenge of fulfilling these criteria to better support and enable data interoperability in their respective domain^10,11^ and European consortia recommend the use of FAIR criteria in health data research^12,13^.

Within the SPHN initiative, the Data Coordination Center (DCC), which is managed by the Personalized Health Informatics group of the SIB Swiss Institute of Bioinformatics, is responsible for designing and implementing a national framework for FAIR research data. SPHN funded a variety of “Driver projects,” which are multisite, collaborative projects in various research fields (e.g., oncology, intensive care, heart disease) that use (routine) health data to answer their research questions. Through the respective data needs of the individual projects, the clinical data and associated metadata requirements in each research area can be determined. Hospital IT personnel, subject matter experts, researchers, and DCC staff work together to achieve data interoperability by precisely defining the semantics of data and storing those semantics and the data in a framework that would be flexible and extensible for covering the needs of existing and future research projects. The broad spectrum of use cases motivated the SPHN to adopt a method with 1) a generic approach composed of a core metadata structure identified to be used in any project and 2) an extension possibility of the core metadata structure by a project with additional metadata as needed where definitions must follow specific rules and conventions. Therefore, the criteria of interoperability and reusability should be ensured not only for the core content between projects but also beyond. Taking these aspects into consideration, SPHN has built a comprehensive ecosystem to support the end-to-end process of making FAIR health data available for research^14^. This data ecosystem is built on a strategy that is divided into three pillars^15^.

The first pillar defines the semantics in the SPHN Dataset, leveraging the domain knowledge of all those involved in the SPHN projects. The SPHN Dataset is built due to the collaboration of the SPHN partners and stakeholders, who provide information about the available data and the needs for research purposes. The SPHN Dataset is based on a compositional approach that includes concepts of general importance for research (e.g., date of birth, administrative gender, diagnosis, drug administration event, procedures, vital signs), concepts relevant for multiple use cases in different SPHN projects (e.g., allergy, adverse event), and concepts of high importance for personalised health research projects (e.g., oncological treatment assessment, tumour stage). In total, the 2022.2 version of the SPHN Dataset includes 63 unique concepts, available in a spreadsheet form (.xlsx format) at https://sphn.ch/document/sphn-dataset/. Each concept is defined and further contextualised with “composedOf” elements (i.e., properties or attributes), which correspond to properties describing that concept. For instance, Allergy, as a concept, is composed of the “reaction type” and the “substance category”, which describe the type of allergic reaction and the category of the substance causing the allergy, respectively. The type of these properties can be other concepts from the SPHN Dataset, specific codes or terms from external standard terminologies, e.g., Systematized Nomenclature of Medicine - Clinical Terms (SNOMED CT, https://www.snomed.org/), local definitions of distinct qualitative values, temporal, quantitative or string values. In principle, SPHN concepts are generalised building blocks that can be used in a variety of contexts. They can be combined into composite concepts, which in turn can be incorporated into more complex compositions. The semantics definition is an iterative process where concepts are defined and refined between the different releases. Further the SPHN interoperability framework provides guidelines for concept design, which allows individual projects to represent their information beyond common data models, e.g., the content of a specific consent form of a registry, which may involve sharing questionnaire data in addition to clinical and genomic data.

The second pillar complements the first one by using standard formalism to represent and store the semantics and its instantiation, i.e., the data. The formalism adopted by SPHN is the Resource Description Framework (RDF, https://www.w3.org/RDF/), an open and flexible standard defined by the World Wide Web Consortium (W3C, https://www.w3.org/). It has been widely adopted in several bioscience projects as a standard framework for data aggregation and exchange to facilitate knowledge building and data exploration^16–20^. RDF is a graph-oriented representation that enables the storage of any data and metadata in a simple and linked structure called “a triple”. A triple is composed of a subject (i.e., the element about which metadata are given in the triple), a predicate (i.e., the type of information provided about the subject) and an object (i.e., the value). Due to its graph-oriented representation, RDF supports implicit documentation of relations between data, which in traditional relational models must be stated explicitly. Hence, RDF is suitable for the representation, storage and exchange of the wide spectrum of information available in health-related data. Linked data also offer the possibility to integrate external terminologies (i.e., medical ontologies and controlled vocabularies), allowing us to represent data in a self-descriptive manner where the meaning of the data is linked to the data itself and can be retrieved easily. Finally, one of the main potentials offered by RDF, and more specifically the Web Ontology Language (OWL, https://www.w3.org/OWL/), is the capacity of representing data using defined logics and applying computer reasoning to discover unidentified, or previously implicit knowledge.

Finally, in the longer term, the third pillar aims to support the integration and translation of this formalism into existing application-targeted models (e.g., RDF to i2b2^21^ translation, RDF to OMOP^22^) to expand applications of the SPHN framework or a project-specific representation that serves the need to answer a given research question.

This manuscript focuses on the implementation of the second pillar and presents how RDF and external terminologies have been used in the context of the SPHN initiative to incorporate SPHN semantics, link clinical data, and provide a way to reach nationwide data interoperability goals in a FAIR manner for personalised medicine research. The goal is to be able to answer competency questions, such as “How many patients have a diagnosis with breast cancer and are undergoing immunotherapy and are alive 2 years after their initial diagnosis?” and “What is the average weight for male patients, above the age of 65, with cardiovascular diseases?”

## Results

### SPHN RDF Schema

**Fig. 1:**
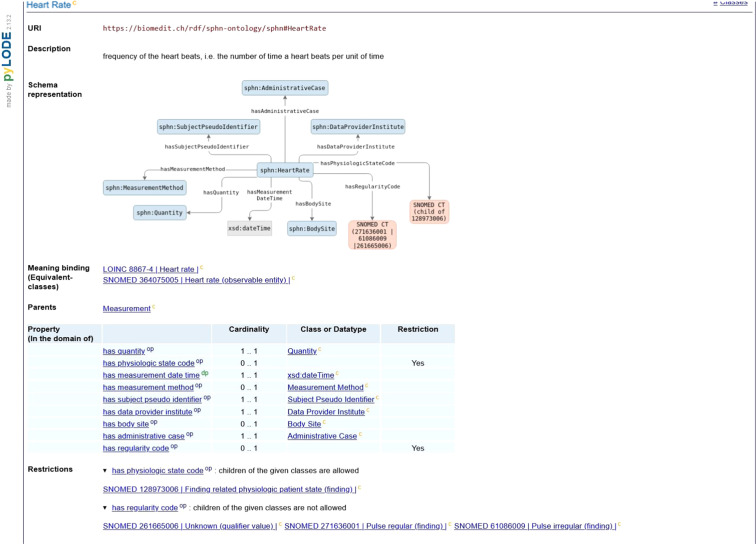
Screenshot of the class Heart Rate from the SPHN RDF Schema visualised in pyLODE. The class Heart Rate is represented with its label, URI (which stands for Uniform Resource Identifier and is a type of IRI that does not support Universal Coded Character Set), description, meaning binding, parent class and connected properties and their restrictions. In addition, a visual schema representation of the Heart Rate class with its possible properties is given.

The SPHN RDF Schema version 2022.2^23^ contains a set of 91 classes and 133 unique properties covering the core data needed for several SPHN research projects (listed below in the Discussion section). The differences in the number of concepts/classes between the SPHN Dataset and the SPHN RDF Schema exist because additional information, not provided in the SPHN Dataset, is defined in the RDF schema, such as administrative metadata (e.g., date of the data release, data provider institute that authored the dataset) and classes grouping a list of individuals (e.g., specialty names of therapeutic areas). Regarding the properties, the SPHN RDF Schema additionally covers links from health-related data to patient, hospital and administrative cases, which are not defined in the SPHN Dataset. The comprehensive, browsable and visualisable documentation of the SPHN RDF Schema based on pyLODE https://github.com/RDFLib/pyLODE is openly accessible at https://biomedit.ch/rdf/sphn-ontology/sphn (see Fig. 1 for a screenshot of the visualisation). In addition to producing a human-readable form of the schema, the pyLODE documentation also provides annotation of best practices, as employed in the SPHN RDF Schema. To further improve the human-readable documentation of the SPHN RDF Schema, pyLODE’s default specification was modified to include the following:

- Custom labels for the SPHN RDF Schema resources and a selection of external resources.
- Every piece of information related to a class (properties, value restriction and cardinalities) are directly provided at the class level.
- Advanced sorting of elements (primary hierarchical and secondary alphabetical).
- Conditional pushdown of inherited properties.
- Various other adaptations and fixes to improve readability.

Furthermore, the server hosting the documentation is configured to provide either human- (HTML) or machine-readable (RDF) content from the SPHN RDF Schema IRI, depending on what is requested, thereby satisfying the requirements of the W3C best practices for publishing RDF vocabularies for both humans and computer applications. The schema is also stored in a git repository, https://git.dcc.sib.swiss/sphn-semantic-framework/sphn-ontology, with additional assets (e.g., SPHN Dataset, SHACL rules, and SPARQL statistical queries). Technical documentation about the entire SPHN Semantic Framework can be found at https://sphn-semantic-framework.readthedocs.io/.

Considering the flexible approach taken by the SPHN, the schema can be extended by the variety of projects according to their needs, following the guidelines specified in the SPHN. In fact, the SPHN core schema is generic and can fit most of the projects’ use cases, but the schemas specific to the projects are the representation of their individual context and vision of the world. To enable and facilitate this extension process, an “SPHN template ontology” turtle file is made available (https://git.dcc.sib.swiss/sphn-semantic-framework/sphn-ontology/-/tree/master/template_ontology), which contains elementary annotations (e.g., import of the SPHN RDF Schema (ontology), imports of external terminologies, import of RDF vocabularies, prefilled project ontology metadata). This template ontology can be opened and used in any ontology editor supporting Turtle as a format in order for projects to extend it with the classes they need (e.g., genomic concepts) and properties (e.g., more detailed meta-data, such as device with which the heart rate is measured) that are not yet provided in the SPHN RDF Schema. Once the projects have extended the schema, the latter is sent to data providers for them to fit data to the schema and send it to the project data users. Project-specific extensions are regularly reviewed by the DCC and reconciled between projects for inclusion in subsequent releases of the SPHN RDF Schema.

### Implementation of the SPHN RDF Schema by data providers

**Fig. 2:**
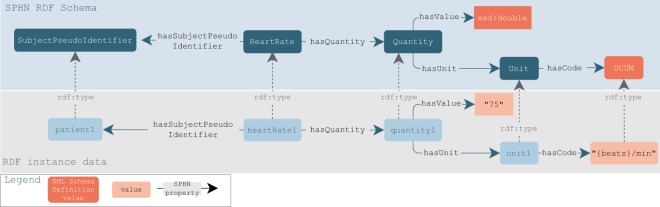
Connection between a subset of the SPHN RDF Schema with a focus on the “HeartRate” concept and an example of data instantiation. In the schema, the top part of the figure shows the SPHN classes and properties in the schema and how they are interconnected. The class “HeartRate” is connected to the class “Quantity” through the property “hasQuantity”, meaning that the heart rate has annotated quantity information, which in turn contains the numerical value (through the property “hasValue”) and the “Unit” of the value (through the property “hasUnit”). In addition, the SPHN RDF Schema links measurements to the patient (i.e., the class “SubjectPseudoIdentifier”) via the property “hasSubjectPseudoIdentifier”. The bottom part of the figure depicts an example of an instance of a heart rate measurement. The heart rate measured (“heartRate1”) from the patient (“patient1”) has a quantity of 75 beats per minute (represented with “beats/min”, as per the UCUM notation). These data instances are connected to the classes in the schema via the property “rdf:type”, which allows annotating the resource with the intended meaning (e.g., patient1 is an instance of SubjectPseudoIdentifier as defined in the SPHN RDF Schema).
- **Step 6: RDF Validation**. The validation of the generated RDF Turtle files is performed using the SPHN quality control framework, which mainly consists of SHACL rules and a set of template SPARQL queries to check compliance with the SPHN RDF Schema and data integrity from local data mart to RDF, respectively.
- **Step 7: Data transfer to secure project space on BioMedIT**. Validated RDF Turtle files are encrypted and transferred to the secure project space (B-space) on the BioMedIT Network^24^ to be accessible and usable by authorised researchers.

The SPHN RDF Schema plays the role of a blueprint for the data providers to fit and map local and hospital-specific data to this schema to achieve semantically and technically interoperable data representations. The current implementation of the RDF schema in the five Swiss University Hospitals involved in SPHN-funded “Driver projects” is specific to each hospital, as it strongly depends on their working processes, development and production infrastructures. However, the various implementations share the following common high-level steps:

- **Step 1: Project data mart creation**. A project-specific data mart, usually consisting of a set of relational views or staging tables (in an SQL sense) and is created by extracting patients and variables from the local clinical data warehouse based on the inclusion and exclusion criteria specified in the research protocol. The local clinical data warehouse centralises clinical data from the various applications of the electronic health record (EHR) system.
- **Step 2: Pseudonymisation and deidentification**. The data in the data mart are pseudonymised (direct identifiers are replaced by project-specific pseudocodes) and deidentified (quasi-identifiers are generalised following a project-specific data deidentification policy which is implemented according to the risk-based SPHN data deidentification concept https://sphn.ch/network/data-coordination-center/de-identification/).
- **Step 3: Local-to-SPHN mappings**. A set of mapping tables or R2RML (https://www.w3.org/TR/r2rml/) files are created, and mappings between each local variable and value set to the classes and properties of the SPHN RDF Schema are defined manually.
- **Step 4: Standardised RDF (SPHN) view creation**. Standardised SPHN views with standardised names are created based on the mappings for a stable mapping definition between the RDF and the source data.
- **Step 5: RDF triples generation and serialisation**. A data conversion script is executed to transform SPHN views into RDF triples. These triples can be materialised in a triple store or serialised into RDF Turtle (.ttl) files for further transfer to external infrastructure. Figure 2 shows an excerpt of the SPHN RDF Schema representation together with examples of data instantiation. The resource “heartRate1” is an instance of “HeartRate” through the property rdf:type), as defined in the SPHN class.

The BioMedIT Network (https://www.biomedit.ch) is a secure IT environment developed in the realm of SPHN for the responsible storage and processing of health-related data as a service. The BioMedIT Network builds on three independent scientific IT competence platforms (the BioMedIT nodes). All three node institutions established a high-performance computing infrastructure in addition to their already existing scientific computing clusters, which was especially designed for processing sensitive research data. B-spaces are BioMedIT secure research project spaces for analysing and exploiting sensitive health data. These environments are each specific to a single research project, which allows project members to collaborate from multiple institutions while also maintaining the security of the data imported for the project.

Currently, pipelines to produce data according to the SPHN RDF Schema are implemented at all Swiss University Hospitals, which are already delivering data to the SPHN research projects (e.g., Swiss Frailty Network and Repository (SFNR)^25^, Personalized Swiss Sepsis Study (PSSS)^26^, Swiss Personalized Oncology (SPO), IMAGINE, Swiss Aging Citizen Reference (SACR); more information on the SPHN projects can be found at https://sphn.ch/network/projects/completed-projects_tiles/). For the largest SPHN project thus far, the Zurich University Hospital (USZ) alone extracted 106 concepts with 475 properties for 5171 patients, resulting in more than 600 million concept instances and more than 2.4 billion triples.

### Use of the SPHN RDF schema by end-users

Once the project data are transferred to the BioMedIT infrastructure, data users exploit the SPHN RDF Schema to understand the structure of the data they receive from data providers and query it accordingly when exploring the data. Mapping the data to the RDF schema requires the process of data instantiation, which asserts that a certain data instance is of a specific type of an SPHN class, thereby describing the semantic meaning of the instance data.

## Discussion

Using RDF, an existing data representation framework, and standards for data storage and exchange is the optimal solution for SPHN to increase the possibility that health-related data will be FAIR, and thus, useful and accessible to researchers. SPHN’s graph-based approach facilitates both data aggregation from different sources and deep data exploration due to existing infrastructures for graph technologies (e.g., data visualisation tools, querying engines, scripting libraries, data reasoning). In addition, the flexibility that the SPHN graph-based approach provides to projects, in terms of expanding the contextual information in the SPHN Dataset (i.e., concepts and properties), is a concrete benefit for projects. Each project can fill possible gaps in the SPHN RDF Schema by enriching it with new concepts and properties to meet the needs of a particular research questions. For instance, the SFNR project needed the definition of variables to facilitate the representation of the defined frailty indices in conjunction with routinely collected patient information. These variables, organised into a taxonomy and represented in RDF, are used in combination with the SPHN RDF Schema to annotate data needed for the project. While this flexibility is valuable, it can also have the disadvantage of making transversal interoperability across projects difficult to achieve, as the content may differ in the specific context. For this reason, the SPHN Dataset and SPHN RDF Schema are iteratively improved with insights added from the different research projects. Before each release, the DCC considers feedback provided by the research projects and incorporates concepts in the SPHN Dataset, which are established to be of general interest. Therefore, in the long term, it is expected that transversal interoperability across disciplines can be achieved as part of the SPHN initiative.

### Benefits of using a semantic-driven framework

**Fig. 3:**
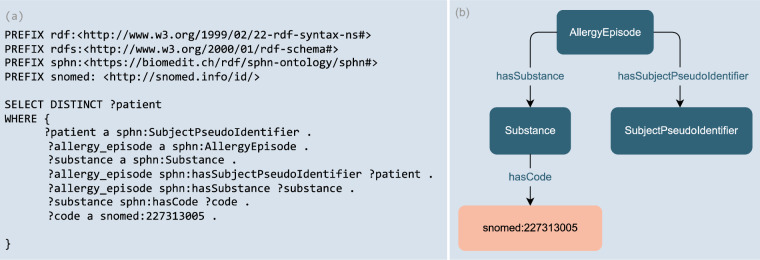
(**a**) SPARQL query and (**b**) visual representation of the related classes. The query retrieves the list of distinct patients (sphn:SubjectPseudoIdentifier) who had an allergic reaction (sphn:AllergyEpisode) to any (including a descendance of) “Pulse Vegetable” substance (snomed:227313005) with RDFS inference enabled.

The real benefit to RDF data users is the ability to access a unified resource of health-related data with all the capabilities that graph technology has to offer. RDF provides increased power and ease for defining and running exploration queries in contrast to relational databases that often require complicated and unintuitive joins. RDF enables knowledge inference possibilities that can reduce the efforts required by data users to explore the data. As a result of mapping (meta)-data to standard terminologies, while some were built as ontologies (e.g., SNOMED CT) or classification with defined hierarchies (e.g., ATC, ICD-10-GM), researchers could simplify their search and obtain contextualised results with simpler queries when compared to SQL. For example, patients may be allergic to many different types of food. In SNOMED CT, peanut, bean and lentils are grouped under the same parent “Pulse Vegetable” (snomed:227313005). If a researcher is interested in querying the data for patients allergic to any type of pulse vegetable, then the query does not need to list all identifiers of the different types of pulse vegetables. Instead, one can simply ask for patients allergic to any type of pulse vegetable (i.e., snomed:227313005) with RDFS reasoning enabled in the triplestore (see Fig. 3 below). This reasoning possibility is enabled and facilitated in RDF due to the fact that graphs have a linked data structure and the contents of the graph follow certain well-defined rules and specifications (e.g., RDFS and OWL). In addition, the flexibility of RDF, as needed for each use case, allows data to be enriched with little effort using existing knowledge sources that are openly available in RDF (e.g., OBO Foundry^27^). In the future, these knowledge sources can be added to SPHN projects to enrich the knowledge made available in datasets.

Finally, using RDF has the potential to enhance the capabilities of machine-learning-based models that can use both data and semantics to infer more nuanced phenotypes and generate better predictions in personalised medicine. For example, the use of graph neural networks enables the creation of patient graph embeddings (i.e., low-dimensional representation of a patient’s data) that can capture curated patient-specific medical knowledge augmented with information from biomedical knowledge repositories and ontologies^28–30^. Several research groups currently mining data for Driver Projects are currently exploring this approaches.

### FAIR principles

As a result of the developed data architecture, the SPHN Dataset combined with the SPHN RDF Schema and data following this knowledge representation fulfil most FAIR principles^8^ (summarised in Table 1). Each class and property established has a unique IRI following the SPHN conventions (FAIR criteria F1), which are published on the BioMedIT website (FAIR criteria F4) and are therefore resolvable on the web through an HTTPS protocol (FAIR criteria A1.1). The SPHN RDF Schema is used as a blueprint for multiple projects, and by publishing the schema centrally, we ensure its long-term availability (FAIR principle A2). Wherever possible, for SPHN concepts, a connection to the IRIs of SNOMED CT and LOINC is provided as meaning binding using “owl:equivalentClass” to improve the understanding of the intended meaning of SPHN data with existing international standards (FAIR criteria I2 and I3). For the property values, either a value set binding to codes from international or national standards (e.g., ATC, ICD-10-GM, LOINC) is defined or SPHN-specific sets of values defined in the SPHN Dataset have been integrated as individuals in the RDF Schema (FAIR criteria I2 and I3). Administrative metadata, such as the date of the data release or the data provider of the dataset, are included in the RDF Schema (FAIR criteria F2 and R1.2) and should be provided in the data. Descriptive metadata, e.g., the method used for a given measurement, are included in the schema linked via RDF properties (FAIR criteria F2 and F3). The SPHN RDF schema is released under the CC BY 4.0 licence (FAIR criteria R1.1) and aspires to meet the best practices defined by the W3C standards as closely as possible (FAIR criteria R1.3). While data in RDF is “formal, accessible, shared, and broadly applicable language for knowledge representation” (FAIR criteria I1), other W3C standards, such as SPARQL, can be used to retrieve data “by their identifier using a standardised communications protocol,” (FAIR criteria A1.1) or SHACL to validate the data generated according to a specific schema. Patient health-related data generated in the context of SPHN are generally not openly accessible due to legal and ethical reasons. Access to the data in the secure BioMedIT project space is controlled via an authentication procedure using the SWITCH edu-ID authentication service (FAIR criteria A1.2). Usage rights, including third-party usage of the data, are explicitly described and controlled in legal agreements, which are settled prior to data sharing between the data providers and the end project users (FAIR criteria R1.1). These agreements should also specify the long-term availability of the data (FAIR criteria A2). Data-providing institutions in SPHN invest heavily in thorough (risk-based) data deidentification. Unless explicitly approved by the responsible ethics committee, only data that have been approved for research by a broad consent of patients (i.e., Generalkonsent) are used in SPHN. In the event of a withdrawal of consent, ongoing projects may be continued by law. A later reuse of the data after consent withdrawal is permitted if the data are subsequently anonymised, i.e., the key to identifying data is deleted in the data-providing hospital and the residual re-identification risk is proved to be low.

**Table 1:** FAIR principles satisfied in the SPHN RDF Schema and data.

FAIR principles	SPHN RDF Schema	SPHN compliant data
F1 Unique identifier	URIs for data elements and for external terminologies	URIs for data instances
F2 Rich metadata	Metadata for data elements	Administrative and descriptive metadata
F3 Linking data and metadata	SPHN RDF properties	SPHN RDF properties
F4 Metadata searchable	Schema dereferenceable on the web (open access)	Administrative metadata is searchable within a project (restricted access)
A1.1 Open communication protocol	HTTPS protocol	SPARQL queries
A1.2 Authentication protocol		SWITCH edu-ID to access the projects B-space on BioMedIT
A2 Long term availability	Available on the website and GitLab	Depends on the data provider and project agreement
I1 Knowledge representation	RDF	RDF
I2 Controlled vocabulary	Meaning binding to SNOMED CT and LOINC Defined list of allowed value sets	Mapping between local codes and standards
I3 Linked data	SPHN and standard RDFS/OWL properties (i.e., rdfs:domain, rdfs:range, owl:equivalentClass, owl:Restriction)	SPHN RDF properties
R1.1 Data usage licence	CC BY 4.0	Individual datasets have their own licence
R1.2 Detailed provenance	Concepts and properties to represent administrative metadata (e.g. extraction datetime, data provider institute)	Administrative and descriptive metadata (e.g. extraction datetime, data provider institute)
R1.3 Community standards	SKOS, Dublin Core (e.g. skos:note, dc:description, dc:title, dct:license)	

### RDF implementation challenges

**Fig. 4:**
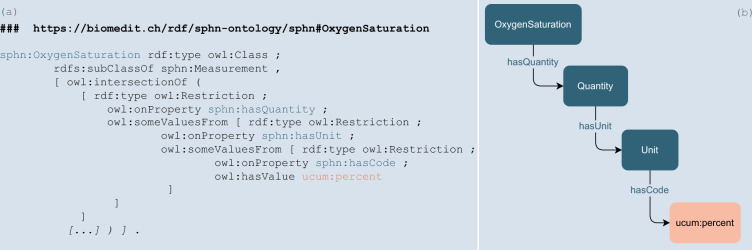
(**a**) Excerpt of the oxygen saturation from the SPHN RDF Schema 2022.2 Turtle (.ttl) format and (**b**) its visual representation. The excerpt shows the implementation of an owl:Restriction for the code allowed for annotating the unit of an oxygen saturation. The only value allowed is “percent” from the UCUM notation, which is stated with the property owl:hasValue.

We developed the SPHN RDF Schema in a multiyear effort over several iterations since 2020, with the first official release published in May 2021. The key features were the definition of naming conventions for classes and properties in accordance with the SPHN Dataset. Domain and range constraints on properties provided the information on the types of classes that are expected for each property. There were still some challenges that were resolved in subsequent releases. For example, initially, it was not possible to represent restrictions on values for a property in a particular property path (e.g., the unit code of an oxygen saturation measurement can only be provided in percent (%)), hierarchical statements were not completely rigorous, and cardinality constraints were not defined. This information was initially represented in RDF as a skos:note (https://www.w3.org/2004/02/skos/). In the 2022 release, the shortcomings of the schema are addressed by a better and controlled way of implementing the semantics. The range constraints on a property are now explicitly defined when the target value is another SPHN class. However, range is not specified when the target value of a property is either a code from an external terminology, an instance of an SPHN code concept (a generic concept used to represent codes from external terminologies that are not in RDF), or SPHN ValueSet (a container for grouping a list of enumerated individuals). Instead, the values that can be used are encoded as an owl:Restriction in the class where this property is used, which solves the issue of encoding value restrictions on a “property path”, as shown in Fig. 4.

With version 2022.2, the SPHN RDF Schema has been improved to encode all the semantics and information of the SPHN Dataset in a controlled and computable manner. This made it possible to develop tools that automatically created SHACLs and SPARQLs for quality control without having to handle exceptions that were not included in the schema in the past.

### Tackling the knowledge gaps

The use of the graph infrastructure comes at a cost: both data providers and data users need to be familiar with the Semantic Web framework. The lack of knowledge about linked data technologies is not uncommon in the clinical domain and therefore requires additional training to understand the SPHN RDF Schema, implement data accordingly, and use RDF to its fullest extent. To help data providers and users gain experience with these technologies, the DCC provides comprehensive documentation and offers in-person and online training on RDF and its related applications (e.g., SPARQL or SHACL) in general and in the context of SPHN.

The goal is that awareness regarding FAIR and linked data will progressively develop in the community. Additionally, data interoperability is not easy to achieve, as it requires large efforts from data providers to deliver data in a standardised way. The reality is that semantic standards are not yet *de facto* fully established in clinical environments. In Switzerland, routine clinical data are often represented in local codes that vary across hospitals and use different national languages, as data collection in a clinical setting is focused on care and not on research. Mapping local codes to standard terminologies is an unavoidable task that must be accomplished to achieve interoperability. The five Swiss University Hospitals involved are moving towards using the SPHN-recommended standards for annotating data provided to SPHN projects. Data mapping is an important task that should not be neglected, as it requires insights from both subject matter experts and standards experts to map the local codes to the correct standard codes. However, this process is currently a manual task that is both time and resource consuming, albeit essential. As in most cases, the standards are not implemented at the source, i.e., in the source IT systems of the hospitals. To support this mapping process, whenever possible, SPHN defines a list of predefined terms to be used for specific metadata. If this is not possible, a recommended standard terminology is given if there is one that fits the data type.

### Future perspectives for the third pillar

The next step of the SPHN semantic interoperability framework is the implementation of the third pillar, which is mainly aimed at facilitating the integration and exploration of SPHN data into other existing platforms to enable broader use of existing data models (e.g., RDF to CSV, RDF to i2b2^31^, RDF to OMOP, RDF to FHIR). Facilitating the use of SPHN data by integrating it with other platforms will further increase interoperability and provide new perspectives on data exploration for interested researchers in Switzerland and abroad^31^.

### Conclusion and outlook

The multidisciplinary community of SPHN developed the SPHN Semantic Interoperability Framework that provides researchers and health care providers with a universal language for knowledge representation of health-related data. The framework builds on existing national and international terminologies and the W3C Semantic Web Stack to conform to most of the FAIR criteria. Using the “words” from various international standard vocabularies (e.g., SNOMED CT or LOINC), with a simple “grammar” (subject-predicate-object expressed in RDF) combined with additional guidelines and rules, SPHN establishes good practices for FAIR data and paves the way for achieving health data interoperability between projects and over time.

In Phase II (2021–2024) of SPHN, the professionalisation of the architecture and further extensions are planned for the development and consolidation of the infrastructure and tool stack to enable data providers and data users to best access and leverage SPHN’s semantic interoperability framework. Furthermore, the launch of the SPHN National Data Streams (NDS; https://sphn.ch/services/funding/nds/) projects will promote and consolidate these infrastructures for data-driven research in Switzerland. The NDS projects include a multiomics layer, which will drive the integration of omics concepts into the SPHN RDF Schema in future releases. As SPHN is a Driver Project of GA4GH, these concepts will be aligned with GA4GH Phenopackets^32^. Since not all information is suited to be represented in RDF (e.g., raw genomic data, imaging), we plan to include a “data file” concept in future releases, which will allow us to connect the meta-data represented in the graph with the raw data files.

## Methods

### Semantic enrichment with existing standard terminologies

To provide standardised and unambiguous meaning to hospital-specific concepts and their properties, and therefore, achieve semantic interoperability, the use of existing international and national standard terminologies is essential. Therefore, we tied concepts and properties (composedOfs) defined in SPHN to two of the most comprehensive reference international medical ontologies: SNOMED CT (https://www.snomed.org/) and the Logical Observation Identifiers Names and Codes (LOINC^33^) where available. This multilevel coding of concepts provides semantic interoperability of the SPHN Dataset along two axes of classification and can be extended to other terminologies based on need in an iterative manner.

In addition, where possible, we defined sets of actual values or codes in the SPHN Dataset to express data instances using appropriate standards and coding systems. The following list of national and international standards are used: the International Statistical Classification of Diseases and Related Health Problems, 10th revision, German modification (ICD-10-GM, https://www.dimdi.de/dynamic/en/classifications/icd/icd-10-gm) for diagnosis, the Swiss Classification Of Procedures (CHOP) for procedures, LOINC for laboratory tests, the Unified Code for Units of Measure (UCUM, http://unitsofmeasure.org) for measurement units, the Anatomical Therapeutic Chemical Classification System (ATC, https://www.whocc.no) for medications, and SNOMED CT for medical concepts. These semantic standard terminologies, mainly available as comma separated value (CSV) files, were translated into RDF-compliant formats (Turtle and OWL/XML) for compatibility with SPHN-compliant data resources (see Table 2). The terminologies provided in RDF have their internationalised resource identifier (IRI) following the pattern https://biomedit.ch/rdf/sphn-resource/<name-of-the-ontology>/<code> and versions are handled with different version IRIs, meaning that one RDF file is generated for each version of the terminology. For terminologies with resolvable weblinks to their codes (i.e., ATC and LOINC), the generated RDF classes use the original IRIs given by the terminology provider. For example, the IRI https://www.whocc.no/atc_ddd_index/?code= is used for ATC codes and https://loinc.org/rdf/ is used for LOINC codes, making their codes resolvable and browsable on the web. Python scripts, accessible at https://git.dcc.sib.swiss/dcc/biomedit-rdf, generate the RDF file for terminologies using the library RDFLib, https://github.com/RDFLib/rdflib, where the codes or terms have been defined as classes (i.e., rdfs:Class), and the human-readable names are defined as rdfs:label and hierarchies; if present in the terminology, they were depicted with the rdfs:subClassOf relation from the child to the parent term. One exception stands with SNOMED CT, which has been directly translated using the Snomed OWL Toolkit, https://github.com/IHTSDO/snomed-owl-toolkit, and the resulting RDF file has been modified and enriched with the ELK reasoner, https://github.com/liveontologies/elk-reasoner, to account for additional annotations required in the context of SPHN. The translated terminologies are available through the BioMedIT Portal, the central access point to the BioMedIT network, a nationwide, secure IT infrastructure established for the mobilisation of health data, and its processing and exploitation for research purposes is in SPHN. The network is accessible via a SWITCH edu-ID login (i.e., a nationwide login system) and the MinIO-based SPHN DCC Terminology Service^34^, developed for facilitating the download process of a bundle of SPHN-supported terminologies by different providers and projects. Further documentation is available at https://sphn-semantic-framework.readthedocs.io.

**Table 2:** List of terminologies made available in RDF.

Terminology	Ontology IRI	Code IRI	Version used in SPHN 2022.2	Versions provided in RDF
ATC	https://biomedit.ch/rdf/sphn-resource/atc/	https://www.whocc.no/atc_ddd_index/?code=	2022	2016–2022
CHOP	https://biomedit.ch/rdf/sphn-resource/chop/	https://biomedit.ch/rdf/sphn-resource/chop/	2022	2016–2022
ICD-10-GM	https://biomedit.ch/rdf/sphn-resource/icd-10-gm/	https://biomedit.ch/rdf/sphn-resource/icd-10-gm/	2022	2014–2022
LOINC	https://biomedit.ch/rdf/sphn-resource/loinc/	https://loinc.org/rdf/	2.72	2.69–2.71
SNOMED CT	http://snomed.info/sct/900000000000207008#	http://snomed.info/id/	2022-02-07	2021-01-31, 2021-07-31, 2022-02-07
UCUM	https://biomedit.ch/rdf/sphn-resource/ucum/	https://biomedit.ch/rdf/sphn-resource/ucum/	2021.1	2021.1

These terminologies are integrated into the SPHN RDF Schema (see below), the technical implementation of the SPHN Dataset, to facilitate data mapping. This not only benefits data providers, who can now link data directly to these formal standards and terminologies, but also data users, who can access all the additional information provided by these standards and add value to the data through a single platform.

### The SPHN RDF schema design and conventions

**Fig. 5:**
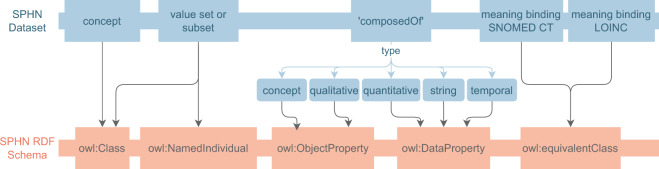
Translation of the SPHN Dataset content into the SPHN RDF Schema. Concepts from the SPHN Dataset are translated into classes in the SPHN RDF Schema. The *composedOfs*, depending on the type of value, are either translated into object properties (i.e., for instances of concepts and qualitative elements) or data properties (i.e., for quantitative, string and temporal elements). Value sets and subsets defined with the SPHN Dataset are translated into either classes (e.g., SNOMED CT, CHOP, LOINC values) or named individuals (i.e., SPHN and UCUM values). Meaning binding to SNOMED CT and LOINC are interpreted as equivalent classes in the SPHN RDF Schema.

The SPHN Dataset aims at delivering semantic information by providing an overview of all concepts and connected semantic standards that are used and shareable within the SPHN. However, it does not aim to provide information on how these different concepts are connected together at the time of data collection. Using RDF, both the semantics defined in the SPHN Dataset and the relations between independent (not semantically linked by composedOf) concepts are represented and stored in a graph structure, where specific concepts can be easily linked to other concepts through the definition of properties (i.e., relations) that convey a precise meaning (see Fig. 5). In SPHN, an RDF schema is generated depicting concepts as “classes” (i.e., owl:Class) and composedOfs as “properties” (i.e., either owl:ObjectProperty or owl:DatatypeProperty, depending on the type of the composedOf). Conventions have been agreed upon to define unique IRIs for classes (e.g., classes are written in Pascal case form) and properties (e.g., all properties start with “has” and are written in camel case form).

To maintain the uniqueness of the concepts defined in the SPHN, the namespace of the SPHN RDF Schema is defined as follows: https://biomedit.ch/rdf/sphn-ontology/sphn. Each RDF version of the SPHN schema is versioned with the year of release and a version number corresponding to the release instance for that year (e.g., https://biomedit.ch/rdf/sphn-ontology/sphn/2022/2 corresponds to the second release of the SPHN RDF Schema in 2022). This preserves the intended meaning of each element across their possible different versions encoded in the schema. In addition, to facilitate data exploration and transformation, hierarchies have been introduced in the schema to group classes with similar meaning (e.g., a measurement class to group concepts, such as body weight, body temperature, and other sorts of measurements) and properties, adding granularity into the schema. The meaning binding of an SPHN class to SNOMED CT and LOINC codes are added as equivalent classes (i.e., owl:equivalentClass). The list of possible codes or terms from external terminologies that are allowed for the annotation of a property value is specified as a restriction (i.e., owl:Restriction) in the class where the property is used. Cardinalities of each property are also embedded in restrictions with the use of owl:minCardinality and owl:maxCardinality. Finally, string-like values from value sets internally defined by the SPHN Dataset and that are not mapped to any preexisting standard are provided as individuals (i.e., owl:NamedIndividual), which makes them available for linking with a unique (SPHN) identifier. The definition of standardised value sets reduces to the absolute minimum the use of uncontrolled string values, guaranteeing the maximum interoperability between datasets following SPHN conventions. The SPHN RDF Schema encodes all information necessary to represent the semantics defined in the SPHN Dataset. These semantic definitions are tag-released as an ontology and provided in Turtle and OWL/XML file formats at https://git.dcc.sib.swiss/sphn-semantic-framework/sphn-ontology.

### Validation and exploration of data compliant with the SPHN RDF Schema

The SPHN RDF Schema specifies the way data must be encoded in the context of SPHN, and data providers follow this specification to transform health data accordingly and provide data to data users. The validation of this type of data can be done through the Shapes Constraint Language (SHACL, https://www.w3.org/TR/shacl/), a W3C standard language built for validating RDF data. In SPHN, SHACL rules are automatically generated in compliance with the SPHN RDF Schema due to an in-house developed tool, the SHACLer (https://git.dcc.sib.swiss/sphn-semantic-framework/sphn-shacl-generator). To complement the SHACLer, the SPHN Semantic Framework provides the quality check tool (https://git.dcc.sib.swiss/sphn-semantic-framework/sphn-rdf-quality-check-tool) that integrates the previously mentioned SHACL rules. However, it also facilitates the validation process by generating a human readable report, which helps to understand possible errors to fix in the data. Furthermore, to support projects in their data exploration step, a set of SPARQL https://www.w3.org/TR/sparql11-query/ queries are produced to gain statistical insights on the data, as well as to extract the metadata of each concept.

## Data Availability

The SPHN RDF Schema is browsable on the web through https://www.biomedit.ch/rdf/sphn-ontology/sphn/2022/2 and available on git at https://git.dcc.sib.swiss/sphn-semantic-framework/sphn-ontology/-/tree/master/ontology and on Zenodo 10.5281/zenodo.7390281^23^.

## Code availability

No custom code was used to generate this work.
